# Walnut shell powder as a low-cost adsorbent for methylene blue dye: isotherm, kinetics, thermodynamic, desorption and response surface methodology examinations

**DOI:** 10.1038/s41598-020-64745-3

**Published:** 2020-05-14

**Authors:** Mohammad Kashif Uddin, Abu Nasar

**Affiliations:** 1 Basic Engineering Sciences Department, College of Engineering, Majmaah University, Al-Majmaah, 11952 Saudi Arabia; 2 Department of Chemistry, College of Science, Majmaah University, Zulfi Campus, Al-Zulfi, 11932 Saudi Arabia; 3 Department of Applied Chemistry, Faculty of Engineering and Technology, Aligarh Muslim University, Aligarh, 202002 India

**Keywords:** Environmental chemistry, Pollution remediation, Pollution remediation

## Abstract

The low cost, eco-friendly and potential biomass, i.e. walnut (*Juglans regia)* shell powder was deployed for the removal of toxic methylene blue dye from contaminated water solution. The important characterization of the waste material was conducted by using several techniques, i.e. Scanning electron microscope, Fourier-transform infrared spectroscopy, Energy-dispersive X-ray spectroscopy, X-ray powder diffraction, Brunauer-Emmett-Teller surface area analysis, and Thermogravimetric analysis. The marked impact of various operating conditions, i.e. dose, concentration, time, pH and temperature on the adsorption process was investigated. Increasing pH resulted in an increase of percent dye adsorption, and the adsorption mechanism was occurred by electrostatic attraction between negative adsorbent surface and positive dye molecules. The equilibrium data suited with Langmuir isotherm model while the adsorption practice followed the pseudo-second-order kinetic model. Higher temperature reduced the adsorption of dye molecules. The adsorption process was spontaneous, exothermic and chemical. The critical statistical analysis of the experimental results was directed by forming the design of the experiment, which was further, optimized by ANOVA, 3D and perturbation plots. The error and predicted values of both the studied responses as derived from the statistical model showed the agreeable results. 0.1 N HCl was found to be effective in complete desorption. The results are very practical and prove the effectiveness of walnut shell powder in the usage of decolorization for methylene blue.

## Introduction

Environmental protection is necessary to conserve precious natural resources such as water. The toxic effect of dye molecules in water is a major environmental issue. Methylene blue (MB) is commonly discharged by several industrial and textile industries in more than the recommended limit^1,2^, which results in water pollution and its dangerous effects. MB (C_16_H_18_ClN_3_S) is an aniline-based basic dye which produces a deep blue color in water or alcohol. It is a greenish blue organic dye which is commonly used as an indicator and stain. It is also used as a synthetic drug for the treatment of methemoglobinemia, psychiatric disorders, nervous system, and malaria^3–5^. At high doses, it can cause anaemia and other series of acute adverse effects. This dye is a noxious product for human beings and may be carcinogenic because of its non-biodegradable nature. The wastewater treatment process is continuously an interesting topic of research for environmentalists. There are numerous ways to treat dye containing aqueous solution such as nanofiltration^6^, photodegradation^7^, coagulation^6^, ozonation^8^ etc. but adsorption method is a convincing process which has lots of benefits^9,10^. Adsorption is a simple, versatile and feasible process. The range of natural and synthetic materials can be used as potential adsorbents for the removal of various water and wastewater pollutants. However, some adsorbents can prove to be economically non-viable because of their low regeneration capacity and not equally effective for many disperse aqueous pollutants^11,12^. Adsorption proves to be a significant method in term of its effectiveness. It is the most famous and highly used process by the scientists worldwide for the removal of different water pollutants^13–15^. There are countless conventional and non-conventional materials which possess excellent adsorptive capacity for organic and inorganic compounds and have been successfully explored^16–19^. Natural waste materials, however, are the attractive and better alternative to synthetic adsorbents due to their large-scale production, cost-effectiveness and safe utilization. The transformation of waste materials into the useful products that can be applied in the industrial process is a fascinating notion to the waste valorization research. Recently, adsorbents made from natural wastes such as cellulose fiber from newspaper waste^20^, waste coffee grounds^21^, Algerian olive cake waste^22^, cotton flower agro-waste^23^, *Luffa aegyptica* peel^24^, spent tea-leaves^25^ were used to remove anionic and cationic dyes from water solution.

Walnut (*Juglans regia*), a drupe nut, belongs to Juglandaceae family are commonly produced in China, United States, Eastern Europe and Iran. The outer skin of raw walnut is a hard, heavy and brown color shell. The underside of walnut fruit is sweet, tasty and edible nut. Walnut nutrition is beneficial for human health and an excellent source of carbohydrate, protein, vitamin C, dietary fibers, fat, and many important vitamin B-complex groups^26^. It has been studied that walnut extracts have antimicrobial, antioxidant^27^ and antibacterial properties^28^ which suggested that it can potentially improve the human’s health. Walnut wood has high tannin content and used to obtain waterproof leather^29^. The raw and modified forms of walnuts have proved their excellent adsorptive capability towards thallium^30^, heavy metal ions^31–33^, organic compounds^34,35^ and dyes^36–39^. The present research aims to use the Box-Behnken design for examining the experimental results obtained by testing the effect of various factors on the proven adsorption performance of raw walnut shell and, for this,a regression equation, ANOVA analysis along with 3D response surface plots and perturbation plots were established. In the present investigation, the adsorption experiments were conducted by choosing methylene blue, one of the most trusted and preferred dye used for experimental studies, as a model adsorbate.

In this study, the efficacy adsorbent made from the walnut shell (WNS) towards the removal of MB dye from aqueous solution has been carried out. The physiochemical characteristics of the walnut shell (before and after adsorption) were determined by important characterization techniques and the effect of various key factors on methylene blue adsorption was tested. In this paper, the experimental investigations of MB adsorption using WNS were first-time statistically optimized by response surface methodology. The adsorbent was successfully excluded the adsorbed methylene blue by passing 0.1 N HCl which proved its good regeneration capacity and practical application in water treatment. The present research shows promising applications in waste and biomass valorization.

## Results and Discussion

### Characterization

#### FTIR

**Figure 1:**
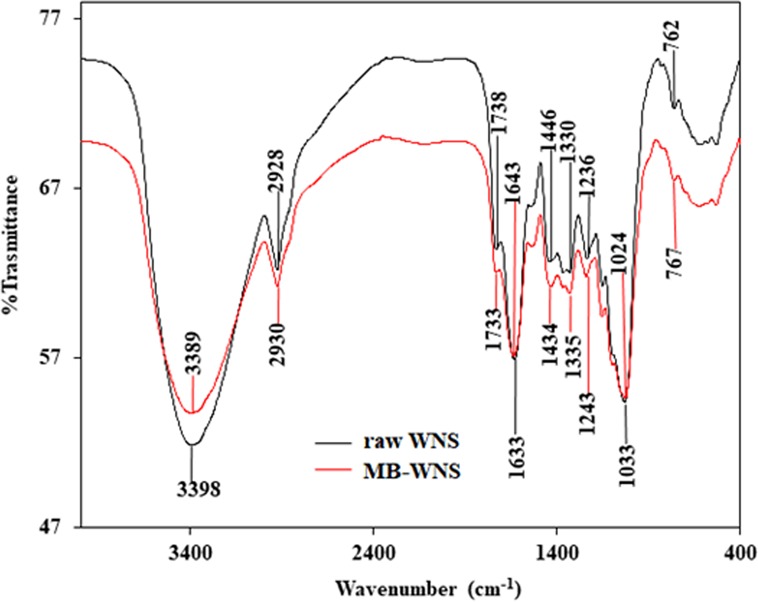
FTIR images of raw WNS and MB-adsorbed WNS.

Figure 1 shows the FTIR of raw WNS and the modifications on its surface after MB adsorption. As seen in figure the major peaks of raw WNS in the functional group region have appeared at 3398, 2928, and 1633 cm^−1^. A broad peeak occured at 3398 cm^−1^ is attributed to the stretching vibration of an O-H, while that of 2930 cm^−1^ is due to C–H stretching vibration of alkane^40^. The band at 1738 cm^−1^ is attributed to C=O stretching of the carbonyl group while the band observed at 1633 cm^−1^ may either be attributed to C=C stretching of alkene or N–H bending of amine^41^. Figure 1 clearly indicates that there is a shift in the position of these bands after the adsorption of MB, which indicates the active involvement of the assigned functional groups. A sharp peak observed at 1643 cm^−1^ in dye loaded WNS is a clear indication of the adsorption of MB. This peak is assigned to the vibrational band of =N^+^(CH_3_)_2_ functional group, which involves in the formation of H-bond^42^. The notable peaks associated with raw WNS as observed at 1446, 1330, 1236, and 1033 cm^−1^ may be assigned to the possible presence of many aliphatic, aromatic, and nitro compounds. The peaks may be assigned to C-C stretching of the aromatic ring, N-O stretching of nitro compound, C-N stretching of aliphatic/aromatic amines^43^. After the adsorption, these peaks are respectively shifted to new positions of 1434, 1335, 1243, and 1024 cm^−1^, which evidently indicates the involvement of the stated groups. The sharp and narrow peak at 1033 cm^−1^ is attributed to the carbohydrate content in WNS^44^. This peak registered a shift to 1024 cm^−1^ after MB adsorption onto WNS. The significant band at 762 and 767 cm^−1^ in both the samples belong to the yellow pigment of WNS^45^. The slight shifting of the characteristic peaks in the FTIR spectrum of MB-adsorbed WNS suggests the successful adsorption of MB on WNS. FTIR study supports the creation of chemical adsorption between functional groups of MB and WNS.

#### SEM and EDX

**Figure 2:**
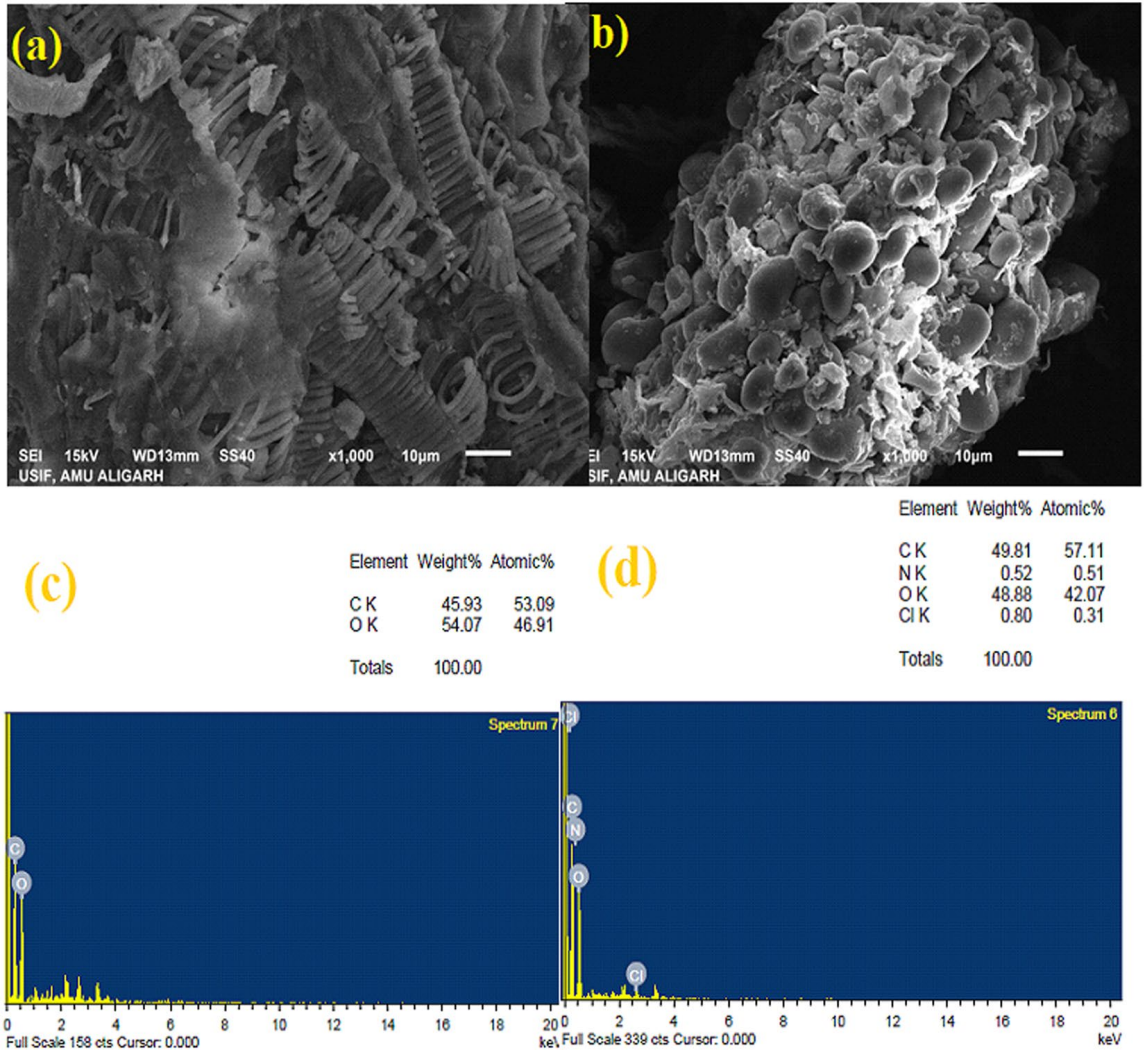
(**a**) SEM image of raw WNS **(b)** SEM image of MB-adsorbed WNS **(c)** EDX spectra of raw WNS **(d)** EDX spectra of raw WNS.

The morphological and surface investigation of raw WNS depicts the thoroughly circular layer of pores in the close arrangement. Figure 2a demonstrates the chain of circular ring arrangement in homogenous, hollow, and hard surface of WNS with high porosity. This proves that the WNS surface was capable of adsorbing MB in its holes and crannies. Figure 2b shows the WNS morphology after MB adsorption. Figure 2b noticeably shows the gathering of MB molecules into the WNS porous structure and the formation of thick assembled attachment of MB to WNS surface. MB molecules covered the WNS surface which can be seen accumulated as spherical like shape onto the material. This confirms the successful MB adsorption onto WNS. EDX analysis was also conducted to detect the elements present in raw and MB-adsorbed WNS. Figure 2c,d shows the elemental compositions of both the samples. The EDX spectrum of raw WNS (Fig. 2c) displays the presence of carbon (C) and Oxygen (O) in WNS. The existence of C (45.93%) and O (54.07%) atoms due to carboxylation prompted the adsorption property of WNS towards MB molecules. Figure 2d indicates the occurrence of nitrogen and chlorine presented in MB onto WNS, which confirms that successful adsorption of MB molecules was carried out.

#### TGA, BET and XRD

**Figure 3:**
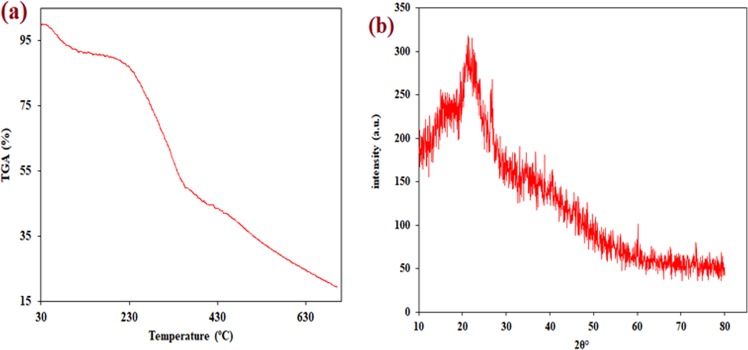
(**a**) TGA analysis of raw WNS **(b)** XRD of raw WNS.

Figure 3a of TGA thermogram of raw WNS displays the multistep decomposition. The cumulative weight loss represents the loss of oxygen and carbon present in WNS. The slow-starting step of weight loss was due to the elimination of free water. The second step registered consistency, the thermal stability of WNS and less decrease of weight loss up to 230 °C which may be due to the decomposition of carbon-based functional groups. The degradation temperature was then started from 250 °C and exhibited rapid weight loss up to 400 °C. After that, the final decomposition can be noted where a maximum component in WNS material was decomposed. The BET surface area, pore volume, and average pore diameter were observed to be 2.505 m^2^ g^−1^, 0.0082 cm^3^ g^−1^, and 13.094 nm, respectively. The XRD pattern of WNS shows a high degree of amorphosity (Fig. 3b). A broad hump is observed in the 2θ range of 20–25° and another peak at ~27°. Apart from these, few diffused peaks are also observed at higher 2θ valves.

#### Effect of pH

**Figure 4:**
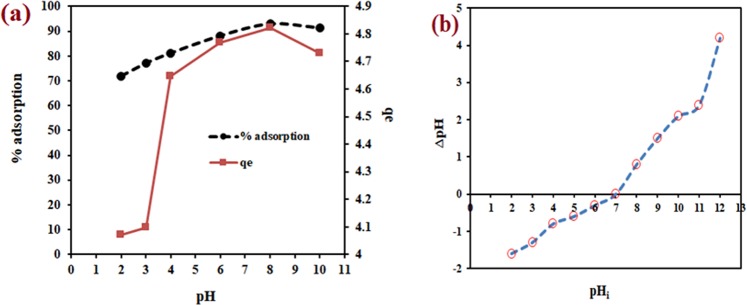
(**a**) Effect of initial pH on percentage (%) MB adsorption onto WNS **(b)** Point of zero charge.

Figure 4a shows the effect of pH on the % adsorption and adsorption capacity of WNS towards MB. The highest adsorption was noted at a basic pH with 93% removal and 4.82 mg/g adsorption capacity. The increase in MB % adsorption was found at pH 2–8 range and then a small fall in pH range of 8–10. The minimum % adsorption and adsorption capacity were observed at pH 2. This is because of the fact that MB is a basic dye, which releases positively charged ions in water. Thus at lower pH values, electrostatic repulsion between the H^+^ ions and protonated MB depress the adsorption of MB onto WNS. In contrast, the basic pH solution has many negatively charged hydroxyl groups that resulted in an increase in MB adsorption and adsorption capacity because of electrostatic attraction. The MB adsorption was favorable in basic solution; hence pH 8 was selected as optimum for further experiments. The point of zero charge (PZC) is a significant concept to find out the pH at which the surface of the material has an equal number of positive and negative charged functions. It helps to understand the adsorption phenomenon and thus the experiment to find out PZC was conducted. The result shows (Fig. 4b) that at pH 7, WNS surface exhibited zero net electrical charge and after that, the surface started to acquire a negative charge. Therefore, the electrostatic attraction at pH> 7 between the negatively charged WNS surface and cationic MB was the reason for the rise in MB adsorption. The PZC result also supports the experimental outcome that pH 8 was the optimal condition of % MB removal.

#### Effect of dose

**Figure 5:**
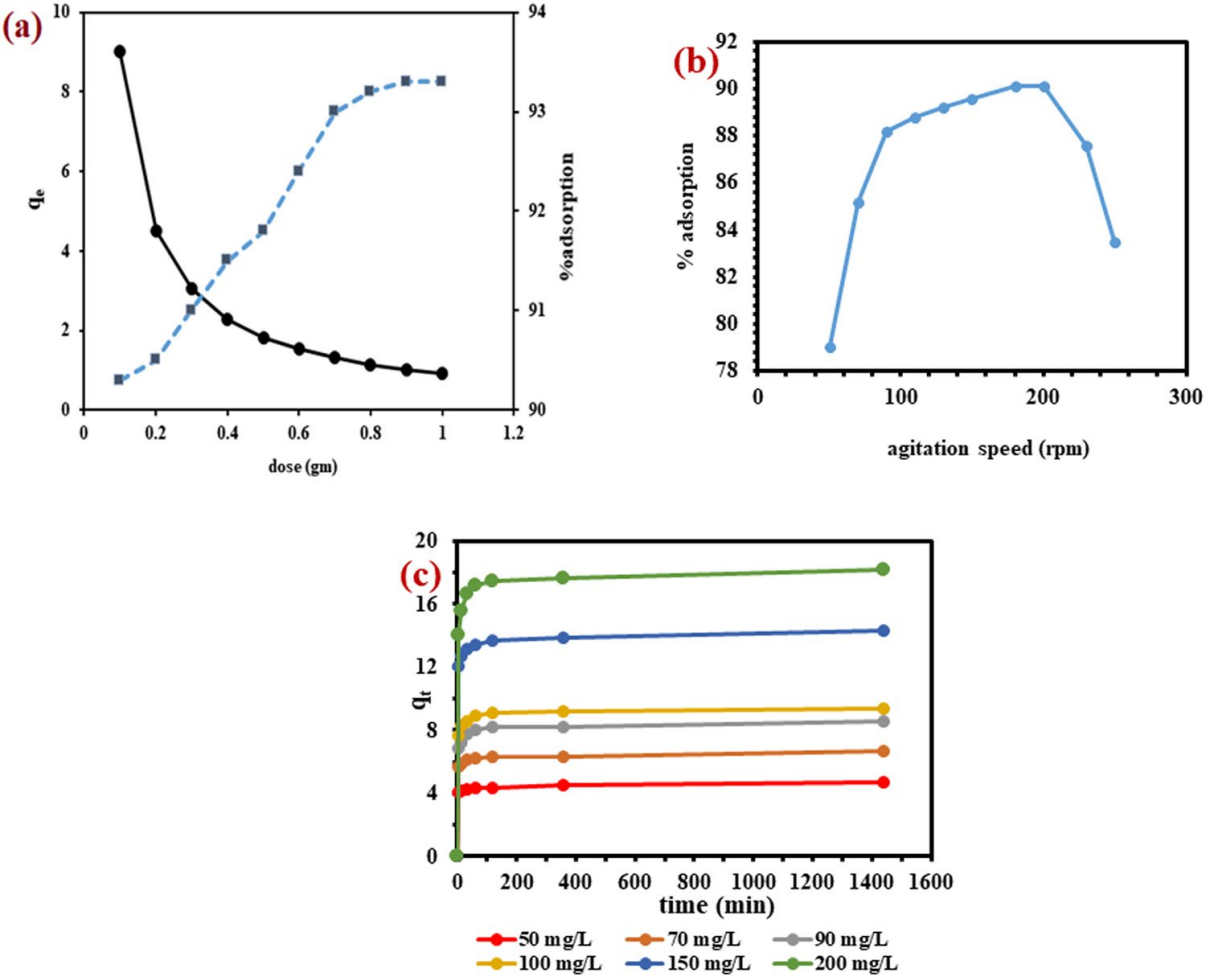
(**a**) The percentage adsorption and adsorption capacity of MB as a function of adsorbent dose **(b)** Effect of shaking speed on % MB adsorption onto WNS **(c)** Effect of contact time and initial concentration on MB adsorption.

The effect of changing the WNS dose (0.1–1.0 g) on MB removal is shown in Fig. 5a. The general trend of increase in % MB adsorption and a decrease in adsorption amount (q_e_) with the increase in WNS dose was observed. Such results are commonly observed in the adsorption system as an increase in dose produces more surface sites lead the way of increase in % adsorption, while it causes a reduction in adsorption amount due to the assemblage of adsorbent mass on the adsorbent surface. The result shows that the high adsorption (90%) happened at the starting low dose of WNS (0.1 g) and after that, there was a slow increase in % adsorption at all studied doses. It means that the active sites of the WNS surface led to the interaction with most MB molecules at a low dose, and then adsorption dose subjected to the small change. Therefore, 0.1 g was chosen as an optimum WNS dose for further experiments as after this dose there was no significant improvement on % adsorption.

#### Effect of shaking speed

Figure 5b shows the results from which it can easily be observed that % MB adsorption increased with an increase in agitation speed and reached a maximum at 200 rpm. This increase was because of the easy diffusion of MB into WNS pores as the kinetic energy of the dye molecules, and adsorbent particles were increased with the increase in agitation speed. After that, the system reached the equilibrium and collision started at highest speed which directed to the decline in % MB adsorption due to detachment of dye molecules from WNS surface. The optimum speed of 200 rpm so obtained was selected for the experiments.

### Effect of contact time and initial concentration

Figure 5c shows the effect of time and initial concentration on the adsorption capacity of WNS for the removal of MB. The rapid increase in the adsorption capacity during the initial stage was observed for each dye concentration. Initially, the adsorption rate was very high due to more availability of active sites of WNS. Later on when the sites were occupied, the adsorption became slow. This slowdown of the rate resulted in the attainment of adsorption equilibrium. The equilibrium adsorption capacity was found to increase (4.713 mg/g to 18.192 mg/g) with the increase in concentration (50 to 200 mg/L) because high concentration created more potential for MB molecules to colloid with active sites on the WNS surface and thus occupied all remaining vacant sites.

### Isotherm

**Figure 6:**
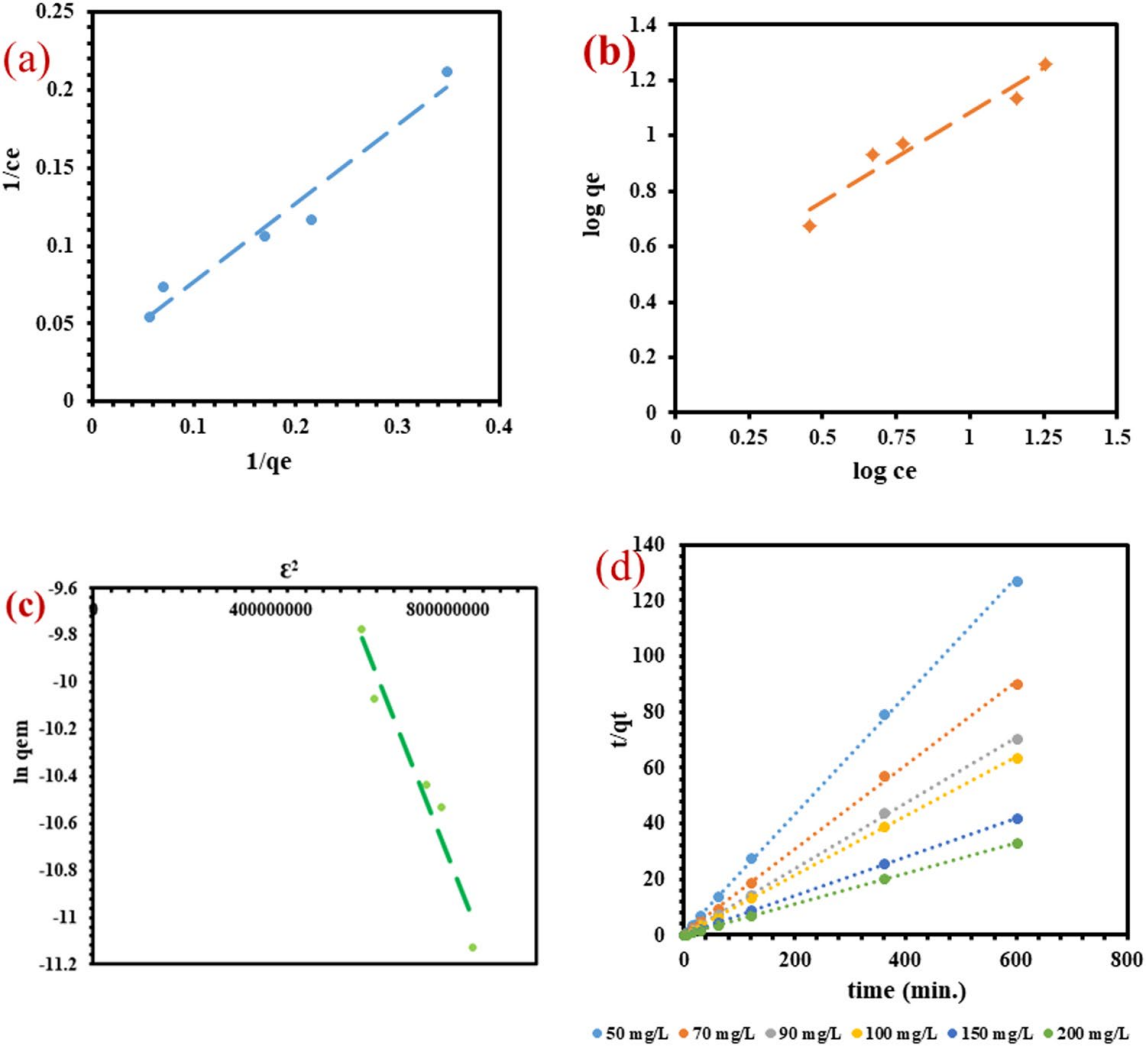
Isotherm plots of the models: **(a)** Langmuir **(b)** Freundlich **(c)** D-R; and **(d)** Pseudo second order kinetics.

Langmuir, Freundlich, and Dubinin-Radushkevitch (D-R) isotherm models are part of mostly adsorption studies because of their great role to investigate the adsorption mechanism of adsorbate on the surface of the adsorbent. The linear equations of these important models are represented in Table 1. The linear trends were observed upon plotting the studied models (Fig. 6a–c) and the parameters belonging to these isotherm models for the equilibrium adsorption of MB onto WNS surface, calculated from the slope and intercept, are also presented in Table 1. It can be observed from the mathematical calculation of the experimental results that Langmuir, Freundlich and D-R isotherms adequately described the adsorption data in a close manner. To compare the results, the statistical analysis was conducted to find out the amount of mean square error (MSE), the total sum of squares (SST), p-value, and standard deviation (*S*_*D*_). It was found from the the error function analysis that the monolayer of MB molecules was formed on the homogenous surface of WNS. Moreover, higher R^2^ values also strengthen the result that Langmuir isotherm was the best-fitted model. The small values of b, K and n confirm the favorable MB adsorption onto WNS. The adsorptive interface between MB and WNS was estimated by mean adsorption free energy (E), whose value can be calculated by the following equation^46^:

1 $${\rm{E}}=\frac{1}{\sqrt{2{\rm{\beta }}}}$$

**Table 1:** Adsorption isotherm parameters and statistical analysis for the adsorption of MB onto WNS.

Isotherm model	Linear equation	Parameters	Values
Langmuir	$\frac{1}{{{\rm{q}}}_{{\rm{e}}}}=\frac{1}{{{\rm{q}}}_{{\rm{m}}}\times {\rm{b}}}\times \frac{1}{{{\rm{C}}}_{{\rm{e}}}}+\frac{1}{{{\rm{q}}}_{{\rm{m}}}}$ Where b refers to Langmuir adsorption constant	b (L/mg) q_m_ (mg/g) R^2^ p-value MSE SST S_D_	0.054 36.631 0.961 0.003 0.000 0.014 0.094
Freundlich	$\log \,{{\rm{q}}}_{{\rm{e}}}=\,\log \,{\rm{K}}+\frac{1}{{\rm{n}}}\,\log \,{{\rm{C}}}_{{\rm{e}}}$ Where K refers to Freundlich constant	K (L/mg)^1/n^ n (g/L) R^2^ p-value MSE SST S_D_	2.762 1.560 0.941 0.006 0.003 0.197 0.277
D-R	$\mathrm{ln}\,{{\rm{q}}}_{{\rm{e}}}=\,\mathrm{ln}\,{{\rm{q}}}_{{\rm{DR}}}-{{\rm{\beta }}{\rm{\varepsilon }}}^{2}$ Where q_DR_ is D-R isotherm saturation capacity and β is D-R isotherm constant which gives mean free energy (E) per molecule of adsorbate when it is transferred from the bulk solution to the surface of the solid and given by Eq. (1). ε is a temperature dependent parameter and related as ${\rm{\varepsilon }}={\rm{RT}}\,\mathrm{ln}\,(1+\frac{1}{{{\rm{C}}}_{{\rm{e}}}})$	β (mol^2^J^2^) E (kJ/mol) R^2^ p-value MSE SST	5 × 10^−9^ 10.00 0.947 0.005 0.018 1.045

If the E value lies between 0–7 kJ/mol, the adsorption process is due to the physical contact between adsorbent-adsorbate interaction and if the value of E falls within 8–16 kJ/mol then the adsorption process comprised with the chemical bonding by ion exchange between adsorbent and adsorbate^47^. As per the D-R parameters, the value of E is 10 kJ/mol which indicates the involvement of a chemical reaction between WNS surface and MB molecules. Thus, it can be concluded from both Langmuir and D-R models that the adsorption mechanism of MB dye molecules on the porous WNS surface was due to the chemical adsorption. The reasonable maximum adsorption capacity (q_m_) of WNS for MB was found to be 36.632 mg/g, which comes out to be superior in comparison to some biomass-based adsorbents like sugarcane bagasse^48^, and Sapindus seeds^49^ which have the q_e_ of 17.434 and 4.610 mg/g, respectively.

### Kinetics

The pseudo-first-order and pseudo-second-order models were used to evaluate the kinetic of MB adsorption on WNS. The pseudo-second-order kinetic plot is shown in Fig. 6d while the parameters of both the models are presented in Table 2. The results indicate that the pseudo-first-order model was unable to precise with the kinetic data. The first-order model displays bad R^2^ values, and the q_e_ calculated came out to be too small to contest with the experimental ones. In contrast, the pseudo-second-order model applied very well through the various concentrations with excellent R^2^ values and good correlation between experimental (q_e(exp)_) and calculated (q_e(cal)_) values. The h values increase with an increase in the concentration because of the availability of more MB molecules and high driving force for mass transfer onto the surface of WNS. The results also support the point that chemical adsorption was involved in binding MB onto WNS. Literature indicates that the removal of MB by many biomass-based adsorbents like citrus limetta peel^13^, rice hull ash^50^, shaddock peel^51^, and wheat straw^52^ were well obeyed pseudo-second-order kinetic model.

**Table 2:** Pseudo-first order and pseudo-second order kinetic parameters for the adsorption of MB onto. WNS.

Concentration (mg/L)	q_e(exp)_ (mg/g)	Pseudo-first-order kinetics			Pseudo-second -order kinetics			
		q_e(cal)_ (mg/g)	k_1_ (1/min)	R^2^	q_e(cal)_ (mg/g)	k_2_ (g/mg/min)	h (g/mg/min)	R^2^
50	4.713	0.647	0.003	0.842	4.697	0.053	1.191	0.999
70	6.668	0.729	0.002	0.466	6.6	0.045	1.988	0.999
90	8.534	0.981	0.004	0.573	8.496	0.04	2.929	0.999
100	9.406	1.021	0.005	0.803	9.398	0.043	3.838	0.999
150	14.363	1.39	0.003	0.64	14.306	0.023	4.842	0.999
200	18.192	1.803	0.003	0.452	18.115	0.021	6.939	0.999

### Thermodynamics

The values of changes in enthalpy (∆H°), entropy (∆S°) and Gibbs free energy (∆G°) accompanying with the adsorption, were determined from the intercept and slope of van’t Hoff plot. The following equations were used to compute the thermodynamic parameters:

2 $$\Delta {\rm{G}}^\circ =-\,{\rm{RT}}\,\mathrm{ln}\,{{\rm{K}}}_{{\rm{c}}}$$

3 $$\mathrm{ln}\,{{\rm{K}}}_{{\rm{c}}}=-\,\frac{\Delta {\rm{H}}^\circ }{{\rm{RT}}}+\frac{\Delta {\rm{S}}^\circ }{{\rm{R}}}$$

Table 3 displays that ∆G° values are negative at all temperatures, which inform about the spontaneity and viability of the adsorption process of MB dye on WNS. The decrease of negative values ∆G° with the increase in temperature indicates that the adsorption process was enthalpy controlled and entropy played an unfavorable role towards the adsorption. The negative values of ∆S° advocate the decrease in disorder at the solid/solution boundary which means that MB molecules became less ordered in solution and rested on WNS surface without making any significant changes in the internal structure of WNS throughout the adsorption process.

**Table 3:** Thermodynamic parameters for the adsorption of MB onto WNS.

ΔH° (kJ/mol)	ΔS° (J/K)	ΔG° (kJ/mol)			R^2^
		303 K	313 K	323 K	
−4.169	−9.118	−1.307	−1.171	−1.124	0.878

### Box-Behnken design

The final regression equations were established for responses Y_1_ (q_e_) and Y_2_ (% MB removal) by fitting the experimental results and the coded factors in the statistical model, which are given below:

4 $$\begin{array}{rcl}R{\rm{esponse}}({{\rm{Y}}}_{1}) & = & 6.25+0.15{{\rm{X}}}_{1}-1.01{{\rm{X}}}_{2}+2.33{{\rm{X}}}_{3}+0.24{{\rm{X}}}_{4}-0.19{{\rm{X}}}_{1}^{2}+0.82{{\rm{X}}}_{2}^{2}\\  &  & +0.085{{\rm{X}}}_{3}^{2}+0.20{{\rm{X}}}_{4}^{2}-2.5\times {10}^{-3}{{\rm{X}}}_{1}{{\rm{X}}}_{2}-2.5\times {10}^{-3}{{\rm{X}}}_{1}{{\rm{X}}}_{3}\\  &  & -0.020{{\rm{X}}}_{1}{{\rm{X}}}_{4}-2.5\times {10}^{-3}{{\rm{X}}}_{2}{{\rm{X}}}_{3}+0.00{{\rm{X}}}_{2}{{\rm{X}}}_{4}+0.083{{\rm{X}}}_{3}{{\rm{X}}}_{4}\end{array}$$

5 $$\begin{array}{rcl}{\rm{Response}}({{\rm{Y}}}_{2}) & = & 89.95+2.46{{\rm{X}}}_{1}+0.35{{\rm{X}}}_{2}+0.94{{\rm{X}}}_{3}+0.69{{\rm{X}}}_{4}-1.51{{\rm{X}}}_{1}^{2}\\  &  & +0.13{{\rm{X}}}_{2}^{2}-0.24{{\rm{X}}}_{3}^{2}-0.49{{\rm{X}}}_{4}^{2}-0.062{{\rm{X}}}_{1}{{\rm{X}}}_{2}+0.00{{\rm{X}}}_{1}{{\rm{X}}}_{3}\\  &  & +2.5\times {10}^{-3}{{\rm{X}}}_{1}{{\rm{X}}}_{4}+0.00{{\rm{X}}}_{2}{{\rm{X}}}_{3}+0.00{{\rm{X}}}_{2}{{\rm{X}}}_{4}-0.94{{\rm{X}}}_{3}{{\rm{X}}}_{4}\end{array}$$

**Figure 7:**
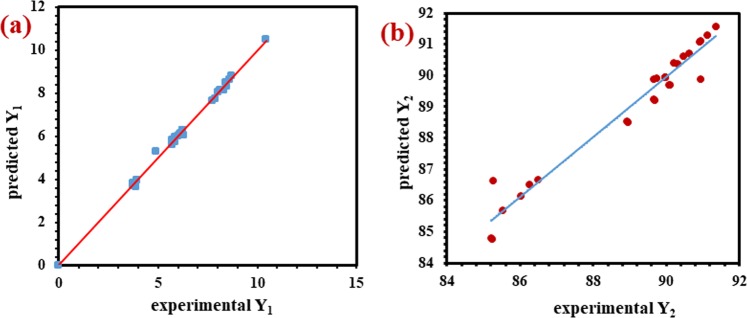
Linear correlation plots between the experimental and predicted values of responses **(a)** Y_1_ and **(b)** Y_2_.

Both the above Eqs. (4 and 5) qualify a good conception of the effects of studied factors and their relations on the responses. Most of the terms in the equations have a positive sign indicate a synergistic effect which means that the factors produced a great combined effect on the response. ANOVA was developed to justify the appropriateness and significance of the quadratic model (Table 4). For responses Y_1_ and Y_2_ the model F-values of 197.356 and 22.90 respectively, and probability> F values of less than 0.05 for both the responses indicate that the model terms were significant for all the studied factors. As per the ANOVA result in case of Y_1_, the terms X_1_, X_2_, X_3_, X_4,_ X_1_^2^, X_2_^2^, X_4_^2^ and X_1_ X_4_ are designated to be significant model terms and in case of Y_2,_ the terms X_1_, X_3_, X_4,_ X_1_^2^ and X_3_ X_4_ were found to be significant. The lack of fit test output the values of 0.43 and 4.82 for Y_1_ and Y_2_ respectively, entails the significance of the model. The adeq. precision values for Y_1_ = 54.556 and Y_2_ = 16.122 are greater than the desirable value of 4 which indicate adequate signal to noise ratio. Moreover, the predicted determination coefficient (“pred. R-squared”) of 0.9710 and 0.7590 for Y_1_ and Y_2_ respectively, are in reasonable agreement with the adjusted determination coefficient (“adj. R-squared”) of 0.9899 and 0.9163 for Y_1_ and Y_2_ respectively, which verified the significance of the model factors. The Fig. 7a,b show the comparison between the experimental and predicted values of responses Y_1_ and Y_2_ respectively. The satisfactory correlation can be seen as the distribution of the data points belongs to the straight line.

**Table 4:** ANOVA results for Y_1_ and Y_2_.

Source	Response (Y_1_)		Response (Y_2_)	
	F-value	p-value	F-value	p-value
Model	197.56	<0.0001	22.9	<0.0001
X_1_	8.66	0.0107	210.46	<0.0001
X_2_	404.03	<0.0001	4.37	0.0552
X_3_	2134.56	<0.0001	31.09	<0.0001
X_4_	22.51	0.0003	16.52	0.0012
X_1_2	7.34	0.0169	43.1	<0.0001
X_2_2	143.59	<0.0001	0.3	0.591
X_3_2	1.55	0.2333	1.12	0.3087
X_4_2	8.47	0.0114	4.56	0.0508
X_1_X_2_	8.197 × 10^−4^	0.9776	0.045	0.8343
X_1_X_3_	8.197 × 10^−4^	0.9776	0	1
X_1_X_4_	5.25	0.038	7.265 × 10^−5^	0.9933
X_2_X_3_	8.197 × 10^−4^	0.9776	0	1
X_2_X_4_	0	1	0	1
X_3_X_4_	0.89	0.3608	10.22	0.0065

**Figure 8:**
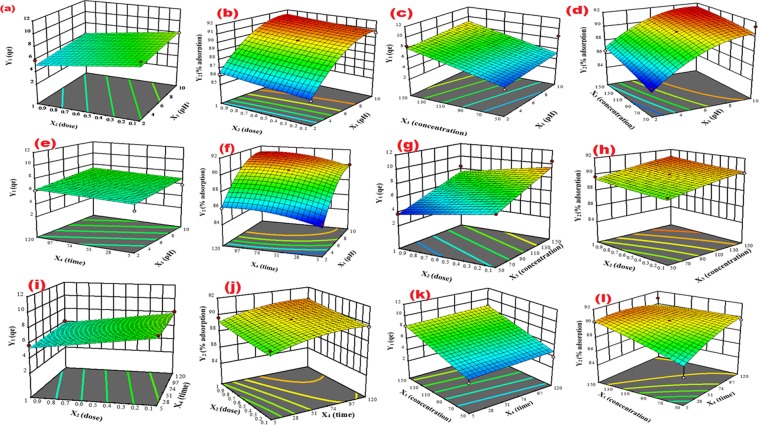
3D surface plots showing the effect of: pH and dose on **(a)** Y_1_ and **(b)** Y_2;_ pH and concentration on **(c)** Y_1_ and **(d)** Y_2_; pH and time on **(e)** Y_1_ and **(f)** Y_2_; dose and concentration on **(g)** Y_1_ and **(h)** Y_2;_ dose and time on **(i)** Y_1_ and **(j)** Y_2_; concentration and time on **(k)** Y_1_ and **(l)** Y_2_.

The three-dimensional (3D) response surface plots illustrate the combined effects of two factors with the responses Y_1_ and Y_2_. Figure 8a,b represent the interaction between X_1_ (pH) and X_2_ (dose) on to the response Y_1_ and Y_2_ respectively. It is evident from the Fig. 8a that Y_1_ (adsorption capacity, q_e_) increased with the increase in X_1_ up to pH 8 and there was a small decrease in q_e _after that. The same effect of X_1_ was found on Y_2,_ hence for this reason pH 8 was chosen as optimum because at low pH values, the MB uptake was weakened due to the competition between H^+^ ions and the adsorption sites. The adsorption capacity (Y_1_) decreased while % adsorption (Y_2_) increased slowly with the increasing adsorbent dose (X_2_) because most of the active sites were utilized at lower WNS dose. Figure 8c,d show the effect of pH (X_1_) and concentration (X_3_) on Y_1_ and Y_2_ respectively. It is evident from the response surface plot that Y_1_ rapidly increased with initial MB concentration (X_3_) because of the adsorption of major portions of MB molecules at high concentration while Y_2_ increased slowly because of the fast adsorption of MB molecules from the starting concentration. Figure 8e,f show the effect of pH (X_1_) and time (X_4_) on both the responses (Y_1_ and Y_2_) respectively. It can be seen that increasing the time (X_4_) of MB adsorption by WNS led to increasing the Y_1_ and Y_2_ values until the subsequent point of equilibrium was reached. Figure 8g,h display the effect of X_2_ and X_3_ on Y_1_ and Y_2_ respectively, and it is clear that increase in X_3_ led to increasing both the responses (Y_1_ and Y_2_) while the increase in X_2_ decrease and increase Y_1_ and Y_2,_ respectively. Figure 8i,j presented the combined effect of X_2_ and X_4_ on Y_1_ and Y_2_ respectively. In the case of Y_1_, the values were decreased and increased with increase in X_2_ and X_4_ respectively, while for Y_2_, both the factors (X_2_ and X_4_) showed an increase in Y_2_. Figure 8k,l show the effect of X_3_ and X_4_ on Y_1_ and Y_2_ respectively and the increase in Y_1_ and Y_2_ was registered with both the factors (X_3_ and X_4_).

**Figure 9:**
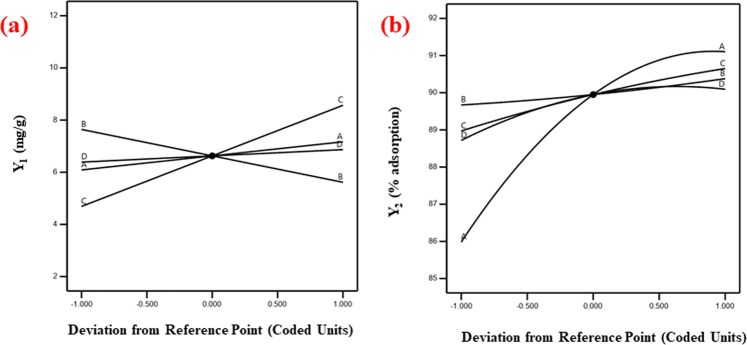
Perturbation plots **(a)** Y_1_ and **(b)** Y_2_.

The perturbation plots examine how the influence of the factors affects the response by comparing the effect of all the factors at a particular reference point. The deviation of the factors from the reference point can be seen in the form of curvature. The more deviation in the factor provides more steep slope while the reasonable flat line shows the insensitivity or less impact of the change in the factor on the response. The perturbation plots were used to analyze the effect of all the studied factors on the responses, Y_1,_ and Y_2_ (Fig. 9a,b). Figure 9a revealed that for Y_1_, the dose (X_2_) and concentration (X_3_) are dominant as compared to pH (X_1_) and time (X_4_) in terms of their influence on the response (Y_1_) which means that Y_1_ is sensitive for X_2_ and X_3._ Figure 9b revealed that for Y_2,_ pH (X_1_) had more influence and mostly affected the response in comparison to other factors (X_2,_ X_3,_ X_4_). It can also be noticed that X_2_ and X_3_ had almost the same effect on the response while X_4_ has not changed the Y_2_ much.

### Desorption and Breakthrough experiment

**Figure 10:**
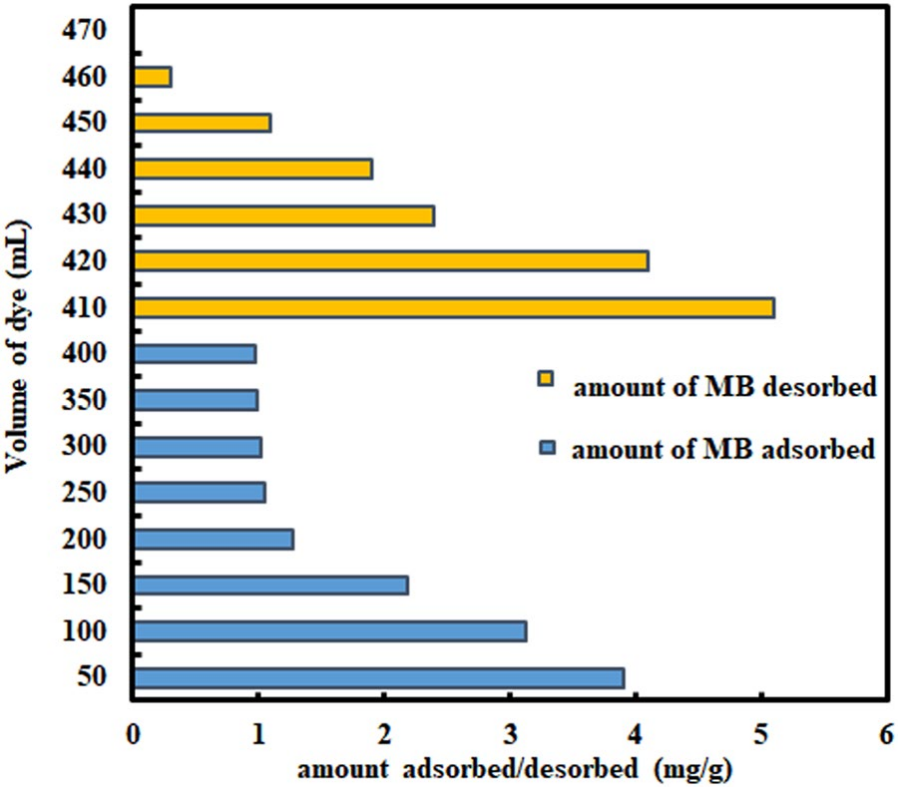
Adsorption/Desorption of MB onto WNS.

**Figure 11:**
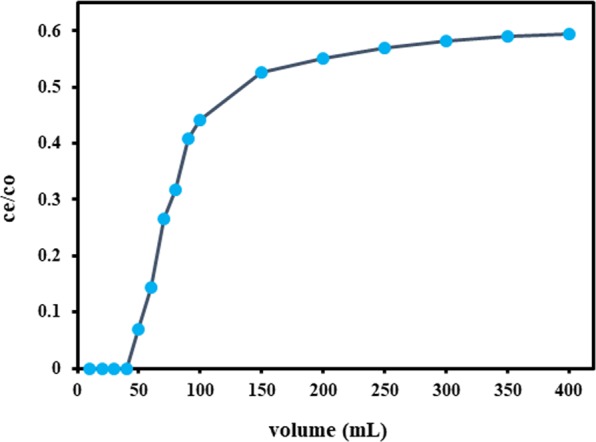
Breakthrough curve.

A volume of 400 mL of MB solution (50 mg/L) was passed through the column which was completely adsorbed at 250 mL run, reached the equilibrium and no marked decrease in amount adsorbed was found after that (Fig. 10). After washing the material in the column, the desorbing solution of 0.1 N HCl was then used. Total 12.54 mg of MB solution was adsorbed after 400 mL run while 11.90 mg was desorbed after 50 mL of 0.1 N HCl and 94.77% of WNS was recovered which can be reused to adsorb MB. The results confirm the wide-ranging application, cost-effectiveness and potential ability of WNS in MB removal from wastewater. The breakthrough curve (Fig. 11) shows that 40 mL of 50 mg/L MB solution was passed undetected and 5 mg/g was the breakthrough capacity at 50 mL of the breakthrough point.

## Materials and Methods

### Adsorbent and Adsorbate

Walnuts were acquired from a local shop of Aligarh city, India and their shells were peeled carefully which were then cleaned by demineralized water and dried in lab oven for the duration of a night at 70 °C. The material was then chopped and deposited into an electric mixer. The ready powdered form of the dried ground biomass was obtained which was then passed through a large sieve, to sort it into different sizes (200–350 µm). Methylene blue dye (A.R. Grade) was bought from a local supplier of (Loba Chemie, India), and used without any distilling process. The demineralized water used to prepare all the solutions for experimental work.

### Characterization

Scanning electron microscopy (SEM) coupled with energy dispersive X-ray (EDX) spectroscopy (JSM6510LV, (JEOL, Japan)) was used to inspect material’s form and structure before and after MB adsorption and also to detect the information of the elemental composition of the adsorbent samples. Fourier transform infrared spectra (Nicolet IS50 Thermo Fisher Scientific FTIR Spectrometer) were perceived to identify the nature of the raw material and the functional groups between MB molecules and WNS after adsorption. The crystallinity of the biomass was observed by X-ray powder diffraction (XRD) analysis were performed by using Bruker AXS Diffractometer. Thermogravimetric analysis (TGA) was conducted in a thermogravimetric analyzer (Perkin-Elmer Pyris) in a nitrogen atmosphere. The BET surface area and pore volume of the sample were measured by BET surface area analyser (Smart Sorb 93). The powdered and dried mass was degassed at 120 °C for 2 h in the regeneration system, and then the studies were performed under the nitrogen adsorption method at 77 K.

### Adsorption procedure

The experiments in three replicates were conducted by batch practice to observe the adsorption possibilities of WNS towards MB dye. All batch adsorption experiments were conducted in brown bottles covered with black paper to avoid any reaction by sunlight. The adsorbent dose of 0.1 gm. was placed in a beaker which contained 50 mL MB of 50 mg/L concentration. The solution was then vibrated at 200 rpm for a noted time, afterwards it was centrifuged at 700 rpm and poured out by the mean of decantation. After that, the residual concentration of MB was measured by a UV–vis spectrophotometer at a pre-optimised λ_max_ of 665 nm. The % adsorption of MB was calculated by the following equation:

6 $$ \% {\rm{adsorption}}=\frac{{{\rm{C}}}_{{\rm{i}}}-{{\rm{C}}}_{{\rm{f}}}}{{{\rm{C}}}_{{\rm{i}}}}\times 100$$

where C_i_ and C_f_ are initial and final MB concentrations, respectively.

The impact of the factors at their different ranges, i.e. pH (2–10), dose (0.1–1.0 gm.) and agitation speed (50–250 rpm) on % MB was investigated. The various isotherm and kinetic studies were completed by altering the concentration (50–200 mg/L) and time (5–600 min.) at optimum pH, speed and temperature. The thermodynamic study was carried out by testing the effect of temperature (30–50 °C) on % MB. The important numerical quantities (parameters) and statistical errors were then determined for all studies.

### Response surface methodology

The response surface methodology of polynomial model was designed by four-factors, three levels (−1, 0 and +1) Box-Behnken design^53–55^ with a total number of 29 experimental runs to optimize the response Y_1_, (MB adsorption capacity of WNS) and response Y_2_ (% MB removal onto WNS) by using design of experiment software. The four factors (X_1_: pH; X_2_: dose; X_3_: initial MB concentration, and X_4_: contact time) were chosen as independent factors (variables) to investigate their significant effects on the adsorption system. Both the responses were fitted in full quadratic order and the results including polynomial regression equations, analysis of variance (ANOVA), predicted responses, three-dimensional, and perturbation plots were obtained by the statistical model (i.e. BBD).

### Desorption

Under the optimum condition, the column study was conducted to find out the desorption and reuse potential of WNS. It was achieved by introducing 0.1 gm. of WNS into the glass wool supported glass column. 400 mL of 50 mg/L MB dye solution was passed through the column at a speed of 1 mL/min. The discharge of the solution from the column was collected in small beakers which were then subjected to the analysis of MB concentration. The exhausted column was cleaned several times by deionized water, and then 0.1 N HCl solution was used as a desorbing agent of adsorbed MB onto WNS. The final discharge was then analyzed. The breakthrough capacity was determined by plotting a breakthrough curve of Ce/Co versus dye volume (mL).

## Conclusion

WNS displayed excellent removal performance toward MB dye. The results from isotherm, kinetic and thermodynamic studies suggested the involvement of chemical bonding between MB molecules and WNS surface during the adsorption process. The optimum parameters for maximum MB adsorption were pH: 8, dose: 0.5 gm, concentration: 50 mg/L, and temperature: 30 °C. The adsorption was spontaneous and exothermic as described by the thermodynamic parameters. The utility of the experiments was successfully optimized by Box-Behnken design. The errors and predicted values for both the responses, as derived from the mathematical model, showed the agreeable results and confirm the favorable effect of the studied factors on MB adsorption by WNS. The complete desorption of the adsorbed MB using 0.1 N HCl showed the viability of WNS. The present work explores the sustainable way to utilize promising waste biomass for industrial wastewater treatment.
